# Influence of anticholinergic drugs on the development of an experimental periodontitis model

**DOI:** 10.25122/jml-2020-0194

**Published:** 2021

**Authors:** Viktor Markiyanovich Batig, Lyudmila Fedorivna Kaskova, Marianna Aleksandrivna Ostafiichuk, Ivanna Ivanivna Abramchuk, Maryia Petrivna Mytchenok, Karina Pavlivna Karatintseva, Mykola Olegovich Ishkov, Iryna Viktorivna Batih, Michael Ivanovich Sheremet

**Affiliations:** 1. Department of Therapeutic Dentistry, Bukovinian State Medical University, Chernivtsi, Ukraine; 2. Department of Pediatric Therapeutic Dentistry,Ukrainian Medical Stemmatological Academy, Poltava, Ukraine; 3. Department of Pediatric Dentistry, Bukovinian State Medical University, Chernivtsi, Ukraine; 4. Department of Foreign Languages, Bukovinian State Medical University, Chernivtsi, Ukraine; 5. Surgery Department No. 1, Bukovinian State Medical University, Chernivtsi, Ukraine

**Keywords:** experimental periodontitis model, generalized periodontitis, lipopolysaccharide, hyaluronidase, trypsin

## Abstract

The endogenous microbiome of the oral cavity plays an essential role in the development of periodontal disease. It also has a significant pathogenic effect on the inner-vation of the oral cavity organs. The experimental determination of the effectiveness of various drugs is required for the effective treatment of periodontal disease, and this involves the creation of a model of experimental periodontitis. The objective of this series of studies was to determine the possibility of reproduction of the experimental model of periodontitis and the study of the effects of anticholinergic drugs on the development of an experimental periodontitis model. The reproduction of the experimental model of periodontitis was performed by injecting the gums of rats with solutions of pathogenic factors: lipopolysaccharide, hyaluronidase and trypsin. We aimed to study the effect of anticholinergic drugs (pilocarpine and atropine) on the development of an experimental model of periodontitis after the injection of a hyaluronidase solution (2 mg/ml) into the rats' gums. The study was performed on white Wistar rats. Elastase activity, malonic dialdehyde content, urease activity (bacterial contamination index), lysozyme activity (an indicator of nonspecific immunity), and catalase activity (an antioxidant enzyme) were determined in the homogenate of the studied tissues. The results of a comparative study of the effect of three pathogenic factors (lipopolysaccharide, hyaluronidase, and trypsin) on the activity of elastase in different tissues of experimental animals (gums, tooth pulp, serum, and gastric mucosa) showed that hyaluronidase has the greatest proinflammatory effect. The action of pilocarpine and atropine was determined with an underline experimental periodontitis model. It was shown that both anticholinergic drugs stimulate the inflammatory process in the periodontium and that anticholinergic drugs enhance the proinflammatory effect of hyaluronidase.

## INTRODUCTION

According to the literature, the microbiome of the oral cavity occupies an important place in the development of periodontal disease [1–4]. This microbiome also has a significant pathogenic effect on the innervation of the organs of the oral cavity [5–7]. The pathogenic action of bacteria is achieved through toxins, among which lipopolysaccharide was the most active [8–10]. Among the toxic microbial factors, there is a number of enzymes that can cause toxic effects due to their catalytic properties, aimed at the destruction of structural biopolymers of the macroorganism. Among such enzymes, the enzyme hyaluronidase [11, 12], which hydrolyzes hyaluronic acid, attracts particular attention. The latter is an intercellular “cement”, and its depolymerization significantly increases the permeability of histo-hematological barriers [13, 14]. This creates a high permeability to the tissues of various substances and bacteria (bacterial translocation) [15–17].

Other pathogenic factors of microbes are proteolytic enzymes that cause degradation of the protein base (collagen, elastin) of vascular walls [18–21]. For the effective treatment of periodontal disease, the experimental determination of the effectiveness of various drugs is required [22, 23]. This involves the creation of an experimental periodontitis model [24–26].

The objective of this series of studies was to determine the possibility of reproduction of an experimental periodontitis model and the study of anticholinergic drugs effect on the development of an experimental periodontitis model.

## MATERIAL AND METHODS

The reproduction of the experimental model of periodontitis was performed by injecting the rats’ gums with solutions of pathogenic factors: lipopolysaccharide, hyaluronidase and trypsin. The drugs were in the form of solutions of 0.9 % NaCl lipopolysaccharide (1 mg/ml), hyaluronidase (2 mg/ml) and trypsin (5 mg/ml), which were injected into the gums in the molar area in an amount of 0.2 ml per rat.

The study was performed on white Wistar rats (45 rats in total). We aimed to study the effect of anticholinergic drugs (pilocarpine and atropine) on the development of an experimental model of periodontitis after the injection of hyaluronidase solution (2 mg/ml) into the gums of rats. In order to achieve this, the rats were previously given oral applications of gels with pilocarpine (2 mg/ml) or atropine (0.2 mg/ml) for two days. The rats were euthanized under thiopental anesthesia (20 mg/kg). Three hours after hyaluronidase injection, the gums and dental pulp were isolated, and blood serum was obtained.

The level of biochemical markers of inflammation was determined in the homogenate of the isolated mucosa: elastase activity and malondialdehyde content, urease activity (total bacterial number), lysozyme activity (an indicator of nonspecific immunity), and catalase activity (antioxidant enzyme) [27]. According to the ratio of the relative activities of urease and lysozyme, the degree of dysbiosis was calculated according to A.P. Levitsky’s approach [28], and the antioxidant-prooxidant index (API) was calculated considering the ratio of catalase activity and malondialdehyde content.

All the results of experimental studies were processed using standard statistical techniques [29].

## RESULTS AND DISCUSSION

Previous experiments have shown that significant pathological manifestations of pathogenic factors are detected after 3 hours. The activity of the proteolytic enzyme elastase was chosen as an indicator of inflammation. It is produced by leukocytes, and the increase of its activity indicates leukocyte infiltration of the studied tissue, which is an important pathogenetic sign of the inflammatory process.

**Figure 1:**
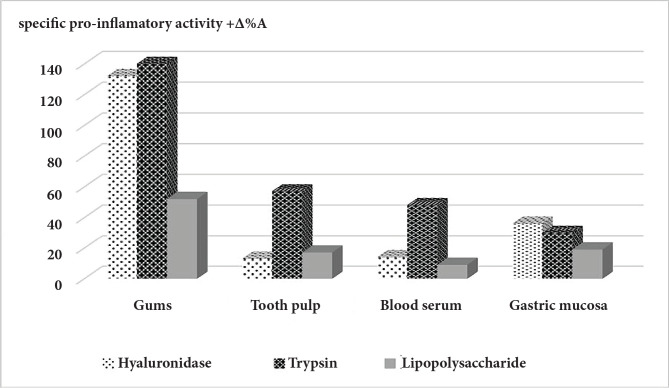
Specific proinflammatory effectiveness of various pathogenic factors.

The results of a comparative study of the effect of three pathogenic factors (lipopolysaccharide, hyaluronidase, and trypsin) on the activity of elastase in different tissues (gums, pulp, serum, and gastric mucosa) are presented in Table 1. According to the obtained data, hyaluronidase has the most significant proinflammatory effect. After recalculating the magnitude of the increase in elastase activity per 1 mg of a pathogen, it was found that hyaluronidase is more effective when acting on the gums, tooth pulp, and serum (Figure 1).

**Table 1:** The proinflammatory effect of pathogenic factors on the level of increased elastase activity in various rats’ tissues (injections into the gums – 0.2 ml for 3 hours).

Medication	Concentration of pathogen mg/ml	Elastase activity, µkat /kg (l)			
		Gums	Tooth pulp	Serum	Gastric mucosa
**Lipopolysaccharide**	**1**				
Control		14.2±1.3	40.4±2.7	115.4±13.3	61.7±2.9
Experiment		32.7±3.1	44.9±6.2	127.7±31.6	83.6±7.5
% increase in activity		+130	+11	+11	+35
**Hyaluronidase**	**2**				
Control		14.2±1.3	40.4±2.7	115.4±13.3	61.7±2.9
Experiment		53.1±2.3	64.4±2.4	172.0±15.6	95.3±2.0
% increase in activity		+274	+59	+49	+43
**Trypsin**	**5**				
Control		14.2±1.3	40.4±2.7	115.4±13.3	61.7±2.9
Experiment		51.1±1.0	70.2±5.7	163.9±4.5	110.7±2.8
% increase in activity		+210	+74	+42	+79

The results of this series of experiments became the basis of the hyaluronidase use for the experimental periodontitis model.

The effect of modulators of the autonomic nervous system (pilocarpine and atropine) on the development of acute experimental periodontitis after the injection of a hyaluronidase solution (2 mg/ml) into the rats' gums was studied in the next series of experiments (15 rats). To perform this, the rats were previously given oral applications of gels with pilocarpine (2 mg/ml) or with atropine (0.2 mg/ml) for two days. Rats were euthanized 3 hours after the hyaluronidase injection, and gums and dental pulp were isolated, and serum was obtained. The biochemical parameters of the rats' gums were determined, and the results are presented in Table 2.

**Table 2:** The effect of oral applications of gels with pilocarpine or atropine on the biochemical parameters of the gums of rats with an experimental periodontitis model.

Indices	Intact rats	Experimental periodontitis model (groups)		
		Control (periodontitis)	+ pilocarpine	+ atropine
		1	2	3
**Elastase, µkat/kg**	47.3±2.4	57.9±1.4p<0.01	52.0±2.9 p>0.05 p1<0.05	51.5±2.3 p>0.05 p1<0.05 p2>0.3
**Malondialdehyde, mmol/kg**	13.1±1.8	10.8±1.0p>0.1	14.9±1.5 p>0.3 p1<0.05	15.2±1.0 p>0.05 p1<0.05 p2>0.3
**Urease, µkat/kg**	0.74±0.10	0.82±0.09p>0.3	0.83±0.16 p>0.3 p1>0.7	0.98±0.11 p>0.05 p1>0.05 p2>0.3
**Lysozyme, IU/kg**	243±11	220±12p>0.05	252±19 p>0.3 p1>0.05	237±17 p>0.3 p1>0.3 p2>0.3
**Catalase, µkat/kg**	8.2±0.2	7.7±0.3p>0.05	7.1±0.1 p<0.01 p1<0.05	8.0±0.2 p>0.3 p1>0.3 p2<0.05
**API index**	6.3±0.4	7.1±0.5p>0.1	4.8±0.3 p<0.05 p1<0.05	5.3±0.3 p>0.05 p1<0.05 p2>0.1

API – antioxidant-prooxidant index. p – compared to group 1; p1 – compared to group 2; p2 – compared to group 3.

The presence of an inflammatory process in the periodontium is evidenced by a significant increase in the activity of elastase (by 22.5%). Applications of gels with pilocarpine or atropine slightly reduce elastase activity, but it remains significantly higher compared to intact rats. Both anticholinergic drugs (pilocarpine and atropine) significantly increase the malondialdehyde content compared to its level in rats with an experimental periodontitis model (control).

Applications of gel with pilocarpine significantly reduced catalase activity and the API index. Gel applications with the proposed drugs did not reduce catalase activity. However, they reduced the API index to some extent. As for the levels of urease and lysozyme, they were not significantly reduced in the experimental periodontitis model after the application of anticholinergic drugs.

## CONCLUSIONS

We performed a comparative study of the effect of three pathogenic factors (lipopolysaccharide, hyaluronidase, and trypsin) on elastase activity in different tissues of experimental animals (gums, tooth pulp, serum, and gastric mucosa), and we found that hyaluronidase has the greatest proinflammatory effect. It has been established that hyaluronidase exceeds the proinflammatory activity of trypsin and even intestinal endotoxin lipopolysaccharide. We also found that anticholinergic drugs enhance the proinflammatory effect of hyaluronidase: they increase elastase activity, malondialdehyde content and significantly reduce catalase activity, API index, alkaline phosphatase activity, and increase acid phosphatase and lysozyme activity.

As a result of the first series of experimental studies, an experimental model of periodontitis was developed using one of the pathogenic effectors of bacteria, namely hyaluronidase, which can significantly increase the permeability of bacteria and their toxins into periodontal tissues.
